# A Readout IC Using Two-Step Fastest Signal Identification for Compact Data Acquisition of PET Systems

**DOI:** 10.3390/s16101748

**Published:** 2016-10-20

**Authors:** Sung-Jin Jung, Seong-Kwan Hong, Oh-Kyong Kwon

**Affiliations:** Department of Electronics and Computer Engineering, Hanyang University, Seoul 133-791, Korea; sj820831@hanyang.ac.kr (S.-J.J.); seongkhong@hanyang.ac.kr (S.-K.H.)

**Keywords:** positron emission tomography, PET, fastest signal identification, readout IC, Geiger-mode avalanche photodiode, GAPD, lutetium-yttrium oxyorthosilicate, LYSO

## Abstract

A readout integrated circuit (ROIC) using two-step fastest signal identification (FSI) is proposed to reduce the number of input channels of a data acquisition (DAQ) block with a high-channel reduction ratio. The two-step FSI enables the proposed ROIC to filter out useless input signals that arise from scattering and electrical noise without using complex and bulky circuits. In addition, an asynchronous fastest signal identifier and a self-trimmed comparator are proposed to identify the fastest signal without using a high-frequency clock and to reduce misidentification, respectively. The channel reduction ratio of the proposed ROIC is 16:1 and can be extended to 16 × *N*:1 using *N* ROICs. To verify the performance of the two-step FSI, the proposed ROIC was implemented into a gamma photon detector module using a Geiger-mode avalanche photodiode with a lutetium-yttrium oxyorthosilicate array. The measured minimum detectable time is 1 ns. The difference of the measured energy and timing resolution between with and without the two-step FSI are 0.8% and 0.2 ns, respectively, which are negligibly small. These measurement results show that the proposed ROIC using the two-step FSI reduces the number of input channels of the DAQ block without sacrificing the performance of the positron emission tomography (PET) systems.

## 1. Introduction

Positron emission tomography (PET) acquires functional images of the human body to analyze the metabolic process, whereas other medical imaging modalities, such as computed tomography [1], magnetic resonance imaging [2], X-ray [3], and ultrasound imaging [4] acquire anatomic images of the human body. The functional image is acquired by measuring the distribution of biological substances labeled with a radiotracer [5]. The radiotracer radiates a pair of 511-keV gamma photons, which result from the annihilation of the electron and positron emitted by the radiotracer, without changing the behavior of biological substances. The pair of 511-keV gamma photons is radiated in the opposite direction and detected by a PET scanner composed of a circular array of thousands of gamma photon sensors such as photomultiplier tubes and Geiger-mode avalanche photodiodes (GAPDs) with scintillators. The readout electronics amplify the output signal of the gamma photon sensor. A data acquisition (DAQ) block then extracts information about the gamma photons, such as position, energy, and time, from the output signals of the readout electronics and use it to find a coincidence pair of gamma photons to create a line of response (LOR). Thus, functional images are constructed of millions of LORs.

To acquire high-resolution images, a PET scanner needs a large number of gamma photon sensors, thereby requiring a large number of readout electronics. Accordingly, the number of input channels of the DAQ block needs to increase in order to extract the required information on the gamma photons from the output signals of the readout electronics. Moreover, since only one coincidence pair occurs at a time, the resources of the DAQ block are wasted to identify the coincidence pair among many gamma photons. To reduce the number of input channels and efficiently identify each coincidence pair, a position decoder circuit (PDC) was reported [6]. The PDC filters out useless output signals of gamma photon sensors by identifying the fastest signal among them. Furthermore, to achieve a high-channel reduction ratio, a high-density PDC was presented by connecting PDCs in series [7]. However, it uses redundant analog delay lines and requires many field-programmable gate arrays (FPGAs). Moreover, these PDCs are too bulky to apply to a PET system with a large number of gamma photon sensors because of complex signal routing and board-level integration. To solve the aforementioned problem, a readout integrated circuit (ROIC) with the fastest signal identification and high-channel reduction ratio is required. Developing such an ROIC is a challenging task because the offset voltage of the comparator in the ROIC causes misidentification of the signal, and, moreover, the fastest signal identification (FSI) circuit requires a high-frequency clock.

In this paper, an ROIC using a two-step FSI is proposed to reduce the number of input channels of the DAQ block by filtering out useless output signals of gamma photon sensors with a high-channel reduction ratio. Self-trimming is employed to reduce the offset voltage of the comparators, and, in addition, an asynchronous fastest signal identifier (AFSI) is adopted to identify the fastest signal without using a high-frequency clock. This paper is organized as follows. Section 2 presents the PET system architecture along with the proposed ROIC. In Section 3, the circuit implementation of the proposed ROIC is described in detail. The experimental results of the proposed ROIC are analyzed and compared with prior works in Section 4. Finally, the conclusions are given in Section 5.

## 2. Overall Architecture

### 2.1. Architecture of a PET System Based on the Proposed ROIC

**Figure 1:**
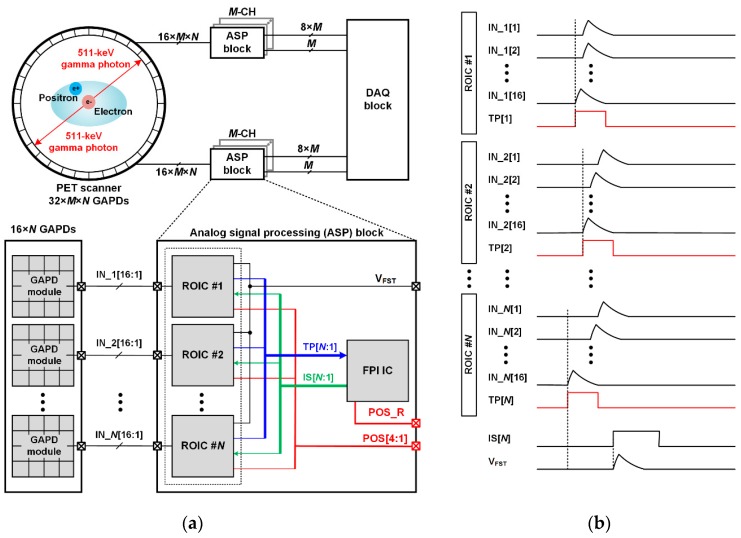
(**a**) Block diagram of a positron emission tomography (PET) system based on the proposed readout integrated circuit (ROIC) and (**b**) timing diagram of the analog signal processing (ASP) block.

Figure 1a shows the block diagram of a PET system based on the proposed ROIC. The PET system consists of a PET scanner, a DAQ block, and analog signal processing (ASP) blocks. The PET scanner is composed of a circular array of thousands of GAPD modules, each of which consists of a 4 × 4 GAPD array. An ASP block consists of *N* ROICs and a fastest pulse identification (FPI) IC. The ASP block identifies the fastest signal among 16 × *N* input signals by using the two-step FSI to reduce the number of input channels of the DAQ block with a high-channel reduction ratio. Each ROIC is implemented, in order to be directly connected to a GAPD module to simplify the signal routing between the ROIC and GAPD module. Figure 1b shows the timing diagram of an ASP block. At the first step of the two-step FSI, each ROIC identifies a local signal, which is the fastest signal among the 16 output signals of its corresponding GAPD module, and generates a timing pulse (TP[*N*]) according to the arrival time of the local signal. In the second step, the FPI IC identifies the fastest timing pulse among TP[*N*:1] to identify the fastest signal among all of the local signals of ROICs in the ASP block. The FPI IC then selects the ROIC that has acquired the fastest signal using selection signals (IS[*N*:1]). The selected ROIC generates a lower four-bit of position data of the fastest signal (POS[4:1]) and transfers the fastest signal (*V*_FST_) to the DAQ block. The arrival time and energy of *V*_FST_ are converted to digital data by using the DAQ block. The rest bits of the position data (POS_R) are generated by the FPI IC. Thus, since only one signal among 16 × *N* input signals is transferred to the DAQ block using multiple ROICs and only a single FPI IC, the number of input channels of the DAQ block is reduced without using complex and bulky circuits, and, moreover, a channel reduction ratio of 16 × *N*:1 is achieved.

### 2.2. Architecture of the Proposed ROIC

**Figure 2:**
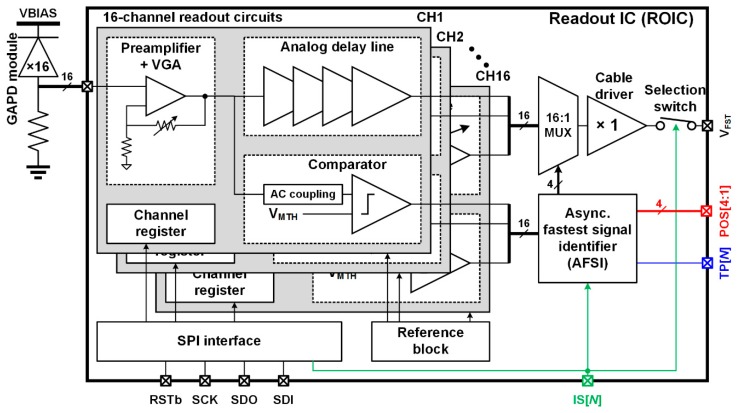
Block diagram of the proposed ROIC.

Figure 2 shows the block diagram of the proposed ROIC which consists of a 16-channel readout circuits, a 16:1 multiplexer, an AFSI, a cable driver, a serial peripheral interface (SPI) block, and a reference block. Each readout circuit consists of a preamplifier, comparator, analog delay line, and a channel register. The preamplifier amplifies the output signal of a GAPD, and the comparator converts the amplified signal to a trigger signal. To identify the local signal, the AFSI finds the fastest trigger signal among the 16 trigger signals of comparators without using a high-frequency clock and generates the timing pulse. When the local signal is identified, the AFSI controls the 16:1 multiplexer to transfer the local signal to the cable driver and transfers the timing pulse to the FPI IC. When an ROIC is selected by the FPI IC using IS[*N*], the selection switch is turned on and the local signal becomes the fastest signal. The fastest signal is then transferred to the DAQ block. The analog delay line prevents distortion of the fastest signal during the two-step FSI by determining a delay time longer than the process time of the two-step FSI. The channel register controls the gain of the preamplifier, the delay time of the analog delay line, and the test mode of each readout circuit. The SPI block reads and writes the channel register. The reference block generates the reference voltage and current, and sends them to the readout circuits.

## 3. Circuit Implementation of the Proposed ROIC

### 3.1. Self-Trimmed Comparator

In the proposed ROIC, the comparator generates the trigger signal with a propagation delay when the output signal of the preamplifier is larger than the threshold voltage. Since the variation in the propagation delay causes an error at the first step of the two-step FSI, it should be minimized. This variation is predominantly affected by the offset voltages of the comparator and preamplifier. To reduce the offset voltage of the preamplifier, an AC coupling is adopted. To reduce the offset voltage of the comparator, auto-zeroing and body bias control are widely used. However, they require additional time to sample the offset voltage, and, moreover, the sampled voltage should be periodically updated due to a leakage current [8,9]. Therefore, a self-trimming scheme is adopted for the comparator to solve the above problem.

**Figure 3:**
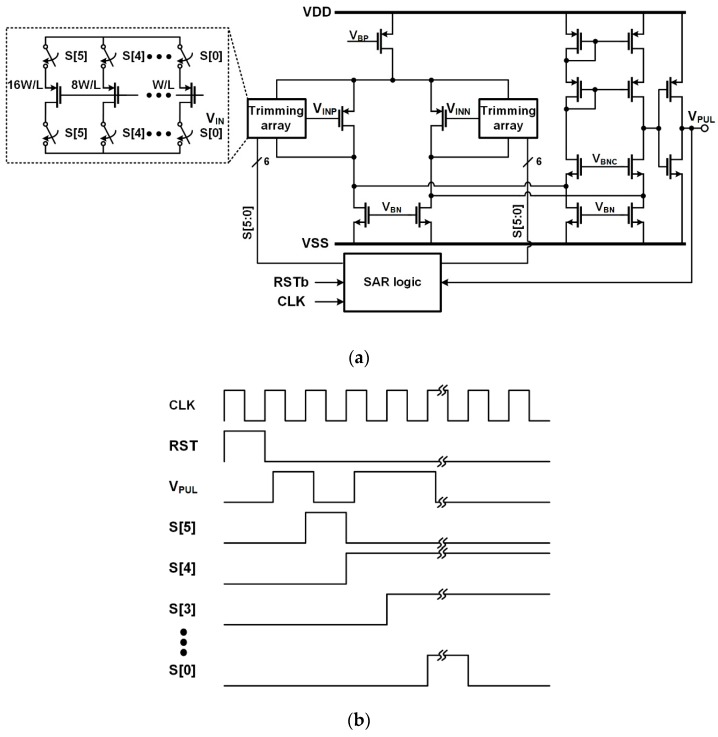
(**a**) Schematic and (**b**) timing diagram of the self-trimmed comparator.

Figure 3a shows the schematic of the self-trimmed comparator, which consists of a differential amplifier, a successive approximation register (SAR) logic, and a pair of trimming arrays. Each trimming array has control switches and six metal-oxide-semiconductor field-effect transistors (MOSFETs) sized in a binary manner. The offset voltage of the comparator is mostly determined by the mismatch of the current factor and the threshold voltage of the input MOSFETs [10]. The current factor is given by

(1) $$\beta =\mu C_{OX}\frac{W}{L},$$

where μ is the carrier effective mobility, *C_OX_* is the gate capacitance per unit area, *W* is the width of the MOSFET, and *L* is the length of the MOSFET. Then, the offset voltage (*V_OS_*) can be expressed as

(2) $$\begin{array}{ll} V_{OS} & =\Delta V_{TH}+\frac{I_{D}}{g_{m}}\left(\frac{\Delta \beta }{\beta }\right)\\ & =\Delta V_{TH}+\frac{I_{D}}{g_{m}}\left(\frac{\Delta \mu }{\mu }+\frac{\Delta C_{OX}}{C_{OX}}+\frac{\Delta W}{W}-\frac{\Delta L}{L}\right) \end{array},$$

where *I_D_* and *g_m_* are the drain current and transconductance of the MOSFET, respectively [10]. Since *V_OS_* is a function of *ΔW*, *V_OS_* can be reduced by adjusting *ΔW* using the trimming array, which is controlled by a successive approximation algorithm.

The timing diagram of the comparator is illustrated in Figure 3b. When the ROIC is turned on, the self-trimming begins after applying the reset signal (RST). Then, *V*_INP_ and *V*_INN_ are tied to the same voltage. Since the offset voltage of the comparator is inherently generated, *V*_PUL_ is in high or low state depending on the polarity of the offset voltage, and one of the two trimming arrays is selected according to *V*_PUL_. Assuming that the offset voltage has a positive value, *V*_PUL_ is high and the trimming array for *V*_INN_ is selected. After the selection, S[5] switches to high and then *V*_PUL_ becomes low, which means that the offset voltage decreases to a negative value. Thus, S[5] is determined to be low. Next, S[4] switches to high and *V*_PUL_ becomes high. The offset voltage decreases but keeps a positive value. Thus, S[4] is determined to be high. This process is repeated until S[0] is determined. Since the determined S[5]–S[0] are stored until the ROIC is turned off, the offset voltage decreases without spending additional time for the update.

**Figure 4:**
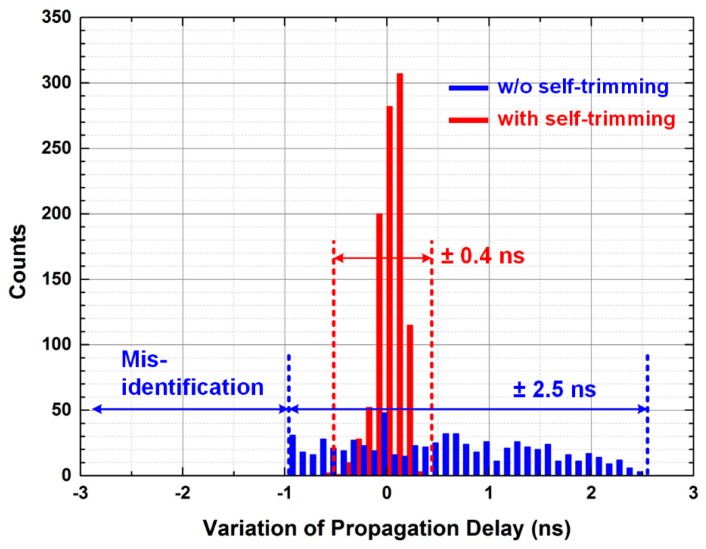
Monte-Carlo simulation results with and without self-trimming.

The 1000 times repeated Monte-Carlo simulation is performed to estimate the variation of the propagation delay, which can cause misidentification at the first step of the two-step FSI. *V*_INN_ is fixed to 0.303 V and *V*_INP_ increases from 0.3 V with a slew rate of 40 mV/ns. When the offset voltage is zero, the propagation delay is 1 ns. When the offset voltage is too large, the output pulse of the comparator is always high. Thus, the identification fails as shown in Figure 4. On the other hand, when the offset voltage is too low, the amplitude of the input signal should be large enough to switch the output of the comparator, thereby increasing the propagation delay. After adopting the self-trimming, the variation in the propagation delay is reduced from ±2.5 ns to ±0.4 ns without the misidentification. Therefore, the maximum propagation delay becomes 1.4 ns, including the variation.

### 3.2. Asynchronous Fastest Signal Identifier

**Figure 5:**
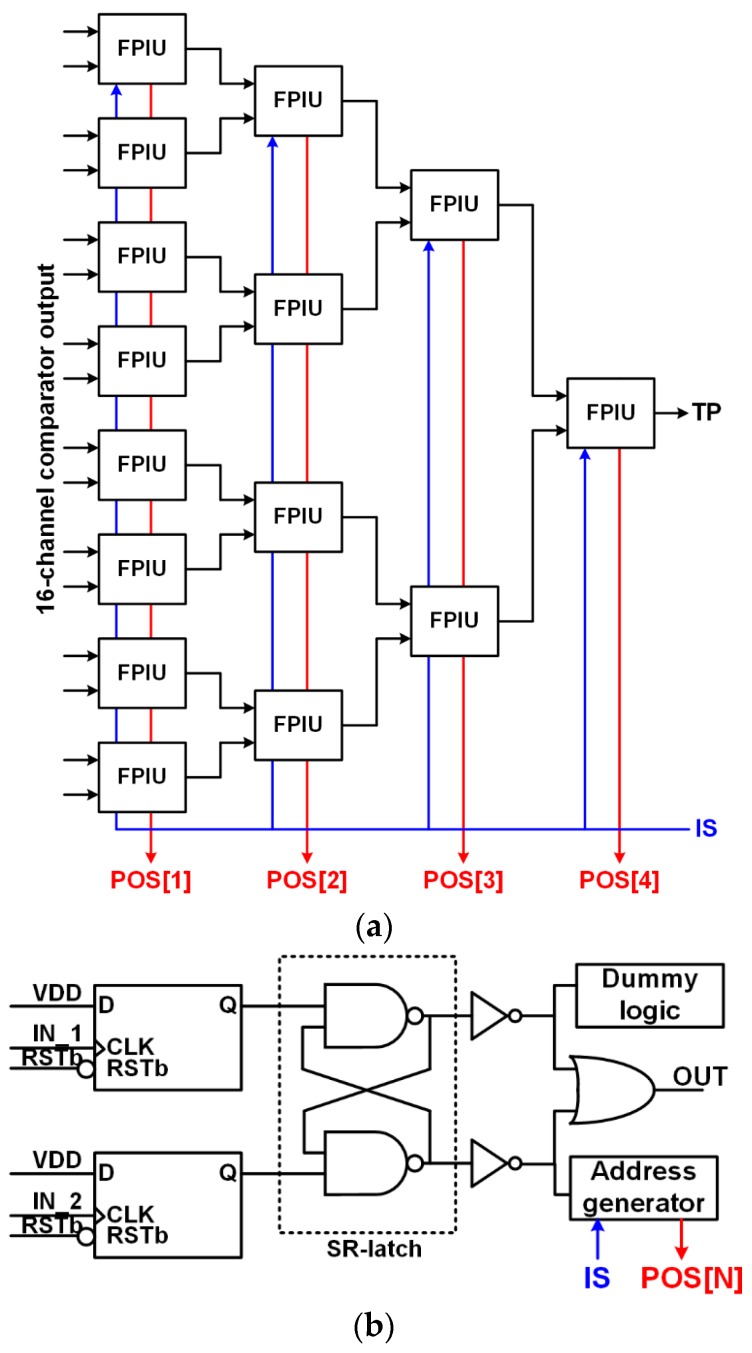
Block diagrams of (**a**) the asynchronous fastest signal identifier (AFSI) and (**b**) the faster pulse identification unit (FPIU).

Figure 5a shows the block diagram of the AFSI, which consists of a four-stage of faster pulse identifier units (FPIUs). The AFSI adopts a tournament structure to minimize the delay difference between output pulses of the comparators. Each FPIU identifies a faster pulse between two input pulses without using a high-frequency clock and generates a one-bit address. Winners of the first stage are transferred to the second stage and competed again to decide which one arrives first at the output of the last FPIU. This process is repeated until the fastest pulse is identified. Thus, the output signal of the last stage becomes a timing pulse.

Figure 5b shows the block diagram of the FPIU. To detect the faster pulse without using a high-frequency clock, two input pulses are applied to the clock terminal of each D flip-flop (DFF), and the set-reset (SR) latch checks which pulse arrives first at the output of DFFs. The faster pulse is transferred to the output of the FPIU through an inverter and OR gate. The address generator generates a one-bit address at each stage when the ROIC is selected by IS. Since a difference in the delay between two input signal paths of the FPIU causes a detection error, the layout of the FPIU is designed to be symmetric, and dummy logic is added to match the load condition of the two inverters.

**Figure 6:**
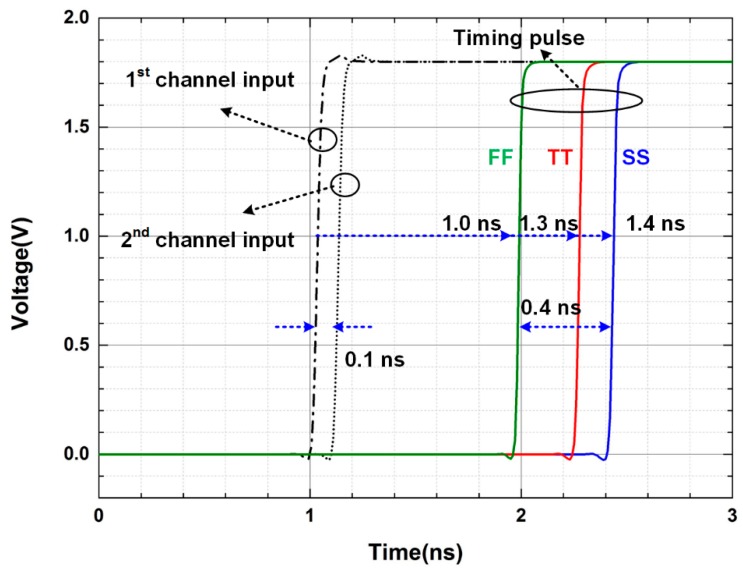
Simulation results of the asynchronous fastest signal identifier (AFSI).

Figure 6 shows the simulation results of the AFSI. Since the AFSI generates the timing pulse, the variation in the decision time could cause the misidentification at the second step of the two-step FSI. Generally, the decision time of the AFSI is influenced by variations in process, voltage, and temperature (PVT). Among the PVT variations, the voltage drop can be ignored because the ROIC does not use a clock during the two-step FSI. In addition, the variation in the decision time caused by the temperature variation is ±0.05 ns, which is negligibly small compared with that due to other process variations. Thus, the variation in the decision time is mainly influenced by the process variation. To estimate the decision time, the simulation according to the process variations in typical, best, and worst cases is performed. A minimum detectable time difference of 0.1 ns is achieved without using a high-frequency clock and the simulated decision time at different process conditions (typical, best, and worst cases) are 1.3, 1.0 and 1.4 ns, respectively. Based on those simulation results, the variation in the decision time (−0.3 ns–0.1 ns) is smaller than the variation in propagation delay (±0.4 ns).

### 3.3. Analog Delay Line 

**Figure 7:**
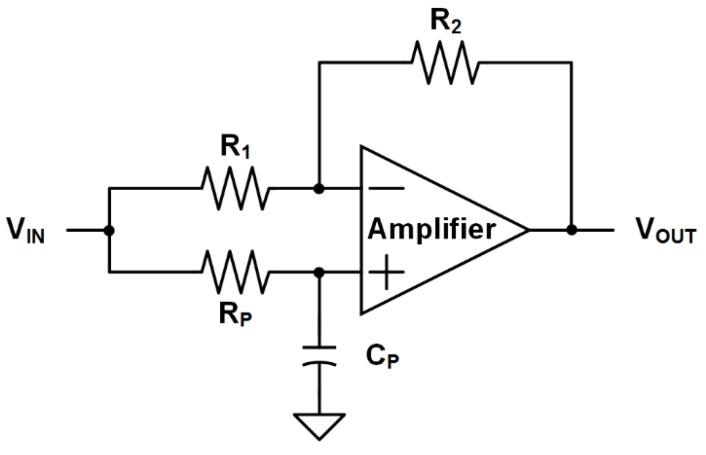
Schematic of the all-pass filter.

To prevent distortion of the fastest signal, the analog delay line should delay the fastest signal during the two-step FSI process. Among various analog delay circuits, an all-pass filter is adopted because it requires neither any sampling operation which could cause sampling harmonics nor a high-frequency clock [11,12,13,14]. The all-pass filter has a unit gain at all frequencies and a phase shift at a designed frequency, which adjust the delay time. Figure 7 shows the schematic of the all-pass filter. To ensure a unit gain, *R*_1_, which is connected to the inverting input of the amplifier, is designed with a resistance value of *R*_2_. *R*_P_ and *C*_P_ determine the phase shift. The transfer function of the all-pass filter, *H*(*s*), can be expressed as

(3) $$H(s)=\frac{sR_{P}C_{P}-1}{sR_{P}C_{P}+1}.$$

The phase shift is determined as follows:

(4) $$∠H(s)=-2\arctan\left(sR_{P}C_{P}\right).$$

To prevent distortion of the fastest signal, the delay time of the analog delay line should be longer than the maximum process time of the two-step FSI, which is the sum of the maximum propagation delay of the comparator (1.4 ns), the maximum decision time of the AFSI (1.4 ns), and the maximum decision time of the second step of the two-step FSI in the FPI IC (20 ns). Thus, the three all-pass filters are connected in series to compose the analog delay line and are designed to have a delay time of 40 ns.

## 4. Experimental Results

**Figure 8:**
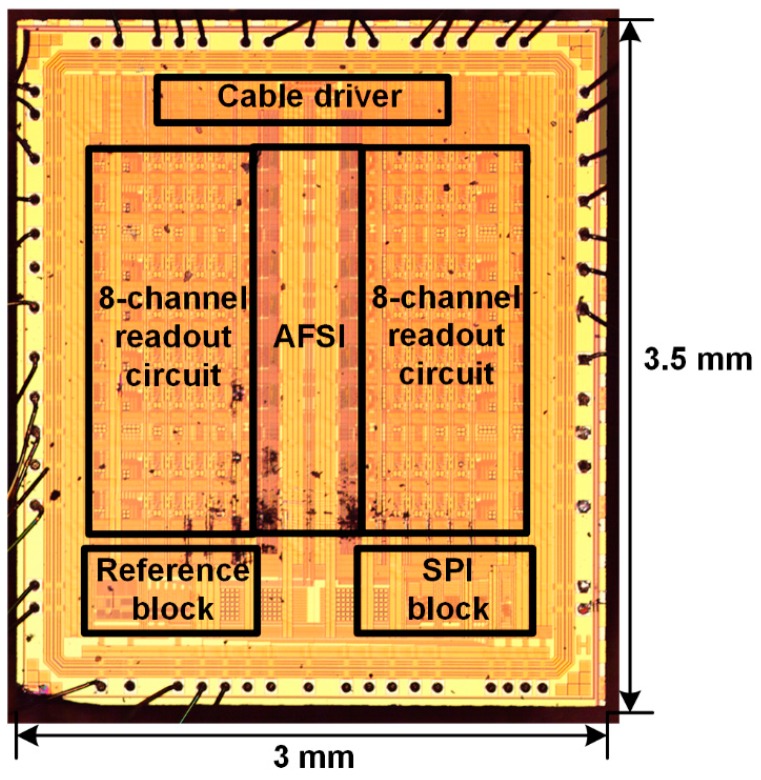
Microphotograph of the fabricated ROIC.

The proposed ROIC was fabricated using a 0.18 µm 1.8 V 1-poly 4-metal complementary metal-oxide-semiconductor (CMOS) process technology. Figure 8 shows a microphotograph of the fabricated ROIC, which occupies an area of 3.5 × 3 mm^2^. The proposed ROIC uses a supply voltage of 1.8 V and consumes a total power of 66 mW, which includes 64 mW of readout circuits, 0.05 mW of reference circuit, and 1.95 mW of cable driver. Since the SPI interface operates only in the initial phase, its power consumption can be ignored. In each readout channel, the preamplifier, analog delay line, and comparator consume 2.8 mW, 1 mW, and 0.2 mW, respectively.

**Figure 9:**
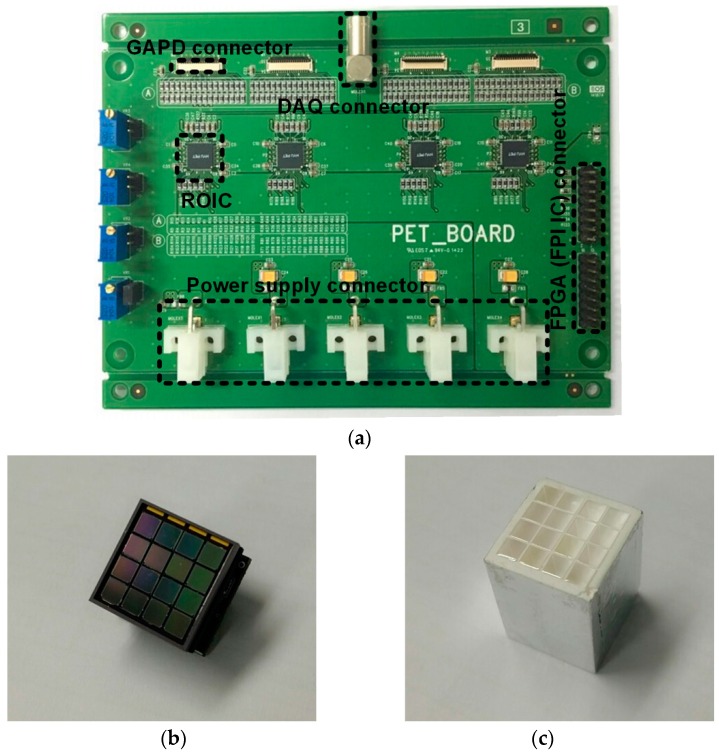
Photograph of a (**a**) test board of the ASP block; (**b**) 4 × 4 Geiger-mode avalanche photodiode (GAPD) array; and (**c**) 4 × 4 lutetium-yttrium oxyorthosilicate (LYSO) array.

Figure 9a shows a photograph of a test board of the ASP block. To measure the performance of the two-step FSI, four ROICs were implemented in the ASP block and the FPI IC was implemented using FPGA. Each ROIC is connected to a 4 × 4 GAPD array (Figure 9b) with a 4 × 4 array of 3 × 3 × 20 mm^3^ lutetium-yttrium oxyorthosilicate (LYSO) scintillators (Figure 9c). An ^22^Na source is used to radiate 511 keV gamma photons. The DAQ block, which has a 100 Mega-sample-per-second ADC, is used to extract the energy and arrival time of the gamma photons.

**Figure 10:**
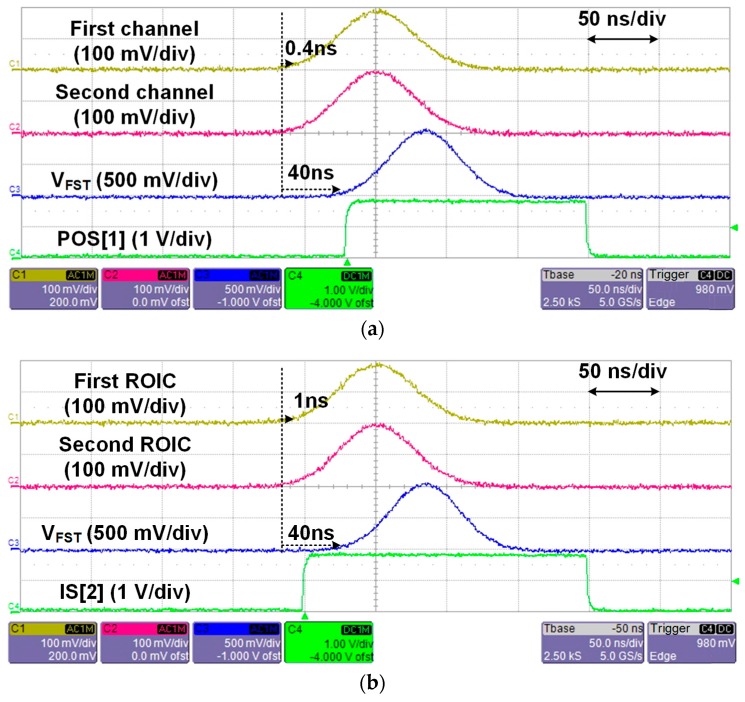
Measured input and output waveforms of the (**a**) ROIC and (**b**) ASP block.

To measure the minimum detectable time difference of the ROIC, two Gaussian pulses with a time difference were applied to the first and second channels of the first ROIC using an arbitrary waveform generator. The time difference was controlled from 0.1 ns to 1.0 ns with a step of 0.1 ns until the faster pulse was identified. The measured results show that the minimum detectable time difference is 0.4 ns. Figure 10a shows the input and output waveforms of the ROIC when the time difference is 0.4 ns. IS[1] was applied to the ROIC and then *V*_FST_ was acquired. Since the Gaussian pulse for the second channel was faster, POS[1] switches to high. The same measurement sequence was repeated for the ASP block, except two Gaussian pulses were applied to the first channel of the first and second ROICs in the ASP block. The measured results show that the minimum detectable time difference of the ASP block is 1.0 ns. Figure 10b shows the input and output waveforms of the ASP block when the time difference is 1.0 ns. Since the Gaussian pulse for the second ROIC is faster, IS[2] switches to high. The difference in the minimum detectable time difference between the ROIC and the ASP block occurs because of variation in the propagation delay of the comparator and the decision time of the AFSI at each ROIC.

**Figure 11:**
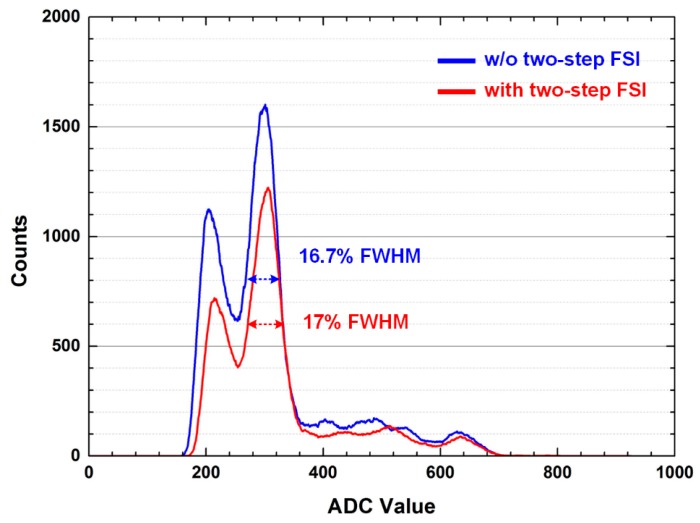
Measured energy spectra with and without the two-step FSI.

**Figure 12:**
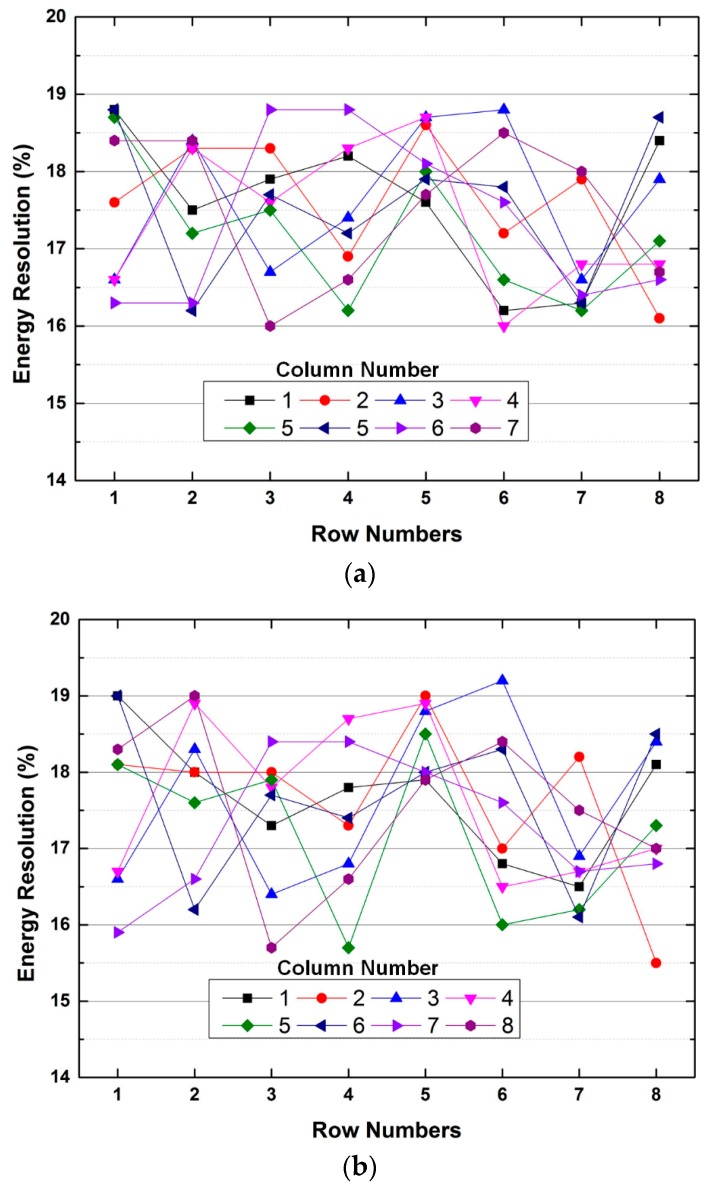
Measured energy resolution (**a**) without and (**b**) with two-step FSI.

The energy resolution, which represents the ability to distinguish between direct and scattered gamma photons, is an important factor of the signal-to-noise ratio and contrast of PET images. It is defined as the full-width at half maximum (FWHM) of a Gaussian fit of the energy spectrum. Figure 11 shows the measured energy spectra of the first channel of the ROIC. The energy resolutions with and without adopting the two-step FSI are 17.0% and 16.7%, respectively. Figure 12 shows the energy resolution of whole channels of the ASP block. The energy resolutions with and without the two-step FSI are 15.5%–19.2% and 16.0%–18.8%, respectively. The difference of the measured energy resolution between with and without the two-step FSI is ±0.6%, which is smaller than the channel variation of 2.8%. A channel variation of 2.8% is mainly caused by the gain variation of the GAPD modules and preamplifiers. Moreover, the gain variation of the analog delay line introduces an additional channel variation of ±0.6% when the two-step FSI is adopted. The channel variation can be reduced further by compensating for the gain variation of the ROIC and adopting the uniform GAPD modules.

**Figure 13:**
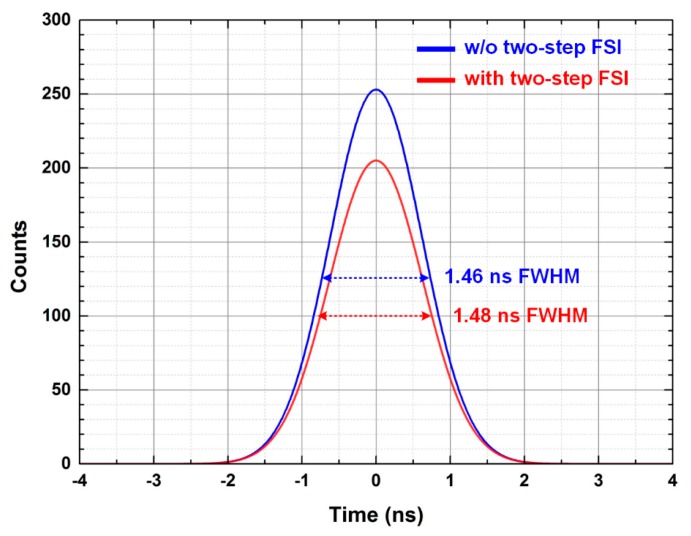
Measured timing spectra with and without the two-step FSI.

**Figure 14:**
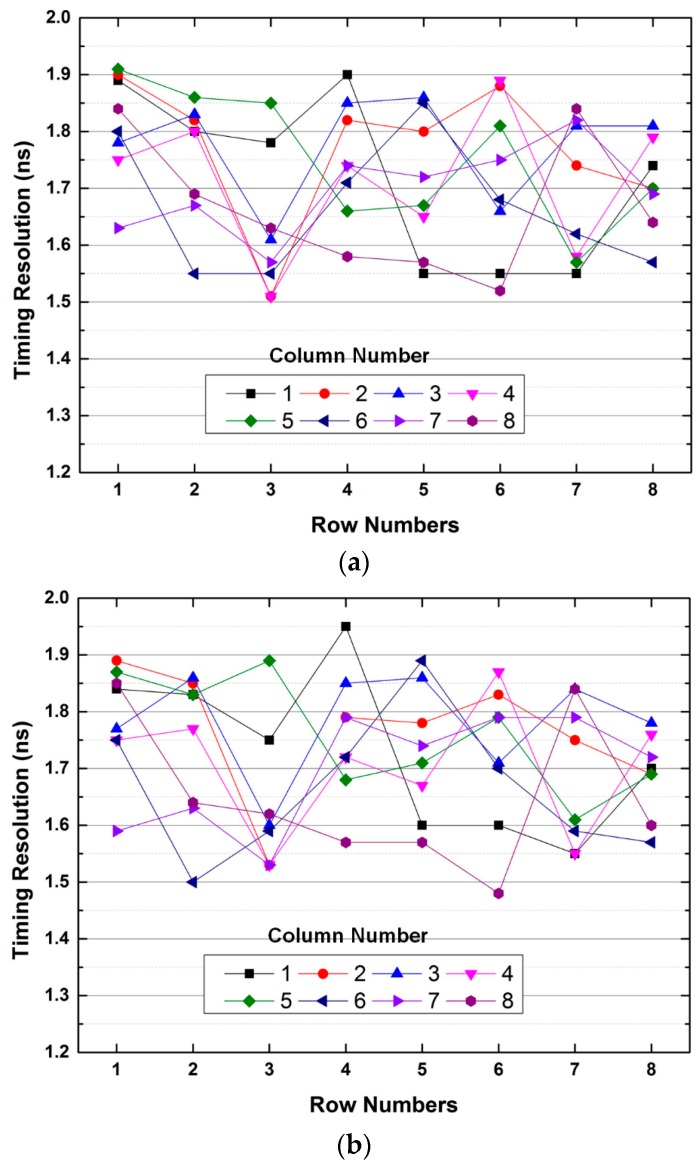
Measured timing resolution (**a**) without and (**b**) with the two-step FSI.

As the timing resolution improves, the statistical noise in PET images decreases, which results in enhancing the quality of PET images. Figure 13 shows the measured timing spectra of the first channel of the ROIC. The timing resolutions with and without the two-step FSI are 1.48 and 1.46 ns, respectively. Figure 14 shows the measured timing resolutions of whole channels of the ASP block with and without the two-step FSI, which are 1.36–1.95 ns and 1.41–1.91 ns, respectively. Thus, the measured timing resolution is comparable to that of commercial small animal PET (2 ns) [15] and human PET (6 ns) [16]. Therefore, the proposed ROIC can reduce the number of input channels of the DAQ block without sacrificing the performance of the PET system.

Table 1 shows a comparison between the proposed ROIC and previously reported works. The proposed ROIC occupies a larger area and consumes more power than other works because of the analog delay line. However, from the system-level point of view, since the number of input channels of DAQ block is reduced by a channel reduction ratio of 16 × *N*:1, the slight increase of the power consumption and area can be compromised.

## 5. Conclusions

In this paper, an ROIC using two-step FSI for PET systems is proposed. The proposed ROIC filters out useless input signals from the PET scanner, and thereby the number of input channels of the DAQ block is reduced by a channel reduction ratio of 16 × *N*:1, where *N* is the number of ROICs in an ASP block. The AFSI identifies the fastest signal without a high-frequency clock. In addition, the self-trimmed comparator prevents misidentification and reduces variation in the propagation delay from ±2.5 ns to ±0.4 ns. The proposed ROIC is implemented with GAPD and LYSO to measure the performance of a PET scanner. The measured energy resolutions with and without two-step FSI are 17.0% and 16.7%, respectively. In addition, the measured timing resolutions with and without two-step FSI are 1.48 ns and 1.46 ns, respectively. These measurement results indicate that the differences in energy and timing resolution with and without adopting two-step FSIs are negligible. Therefore, the proposed ROIC reduces the number of input channels of the DAQ block of PET systems without sacrificing performance of PET systems.

## Figures and Tables

**Table 1:** Comparison with previously reported works.

Parameter	This Work	[17]	[18]	[19]
**Process**	0.18-μm CMOS	0.35-μm CMOS	0.35-μm CMOS	0.35-μm CMOS
**Supply voltage**	1.8 V	3.3 V	3.3 V	3.3 V
**Detector module**	LYSO/GAPD	LSO/APD	LYSO/MCP-PMT	CZT
**Signal reduction**	Two-step FSI	N/A	N/A	N/A
**Channel reduction ratio**	64:1 (16 × *N*:1) *	N/A	N/A	N/A
**Min. detectable time difference**	1 ns	N/A	N/A	N/A
**No. of channels**	16	16	10	8
**Power consumption**	4 mW/channel	10 mW/channel	15 mW/channel	3 mW/channel
**Chip area**	3 × 3.5 mm^2^	2.5 × 1.7 mm^2^	2.8 × 2.18 mm^2^	2.28 × 2.28 mm^2^

* *N* is the number of ROICs in the ASP block.
